# Gallbladder Volvulus in a Patient with Type I Choledochal Cyst: A Case Report and Review of the Literature

**DOI:** 10.1155/2016/5626531

**Published:** 2016-09-22

**Authors:** George Younan, Max Schumm, Fadwa Ali, Kathleen K. Christians

**Affiliations:** Division of Surgical Oncology, Department of Surgery, Milwaukee, WI, USA

## Abstract

*Introduction.* Gallbladder volvulus is a rare, potentially fatal condition unless diagnosed and treated early. Choledochal cysts are rare congenital malformations of the biliary tree predisposing to different pathologies and posing the risk of degradation into cholangiocarcinoma and gallbladder cancer. Dealing with both diseases at once has not been published yet in the literature.* Presentation of Case.* We report a case of gallbladder volvulus in an elderly female who happened to have a concomitant type I choledochal cyst. Treatment was achieved with a cholecystectomy and observation and follow-up of the choledochal cyst.* Discussion.* Prompt diagnosis and surgical management of gallbladder volvulus is important to avoid the morbidity and mortality of gangrenous cholecystitis and biliary peritonitis in a frail old population of patients. Precise clinical diagnosis, supplemented with specific imaging clues, helps in the diagnosis. Management of choledochal cysts is also surgical; however the timing of surgery is still a matter of debate.* Conclusion.* We describe in this report the first case of gallbladder volvulus in a patient with a choledochal cyst and propose a management algorithm of a very rare biliary tree pathology combination.

## 1. Introduction

Gallbladder volvulus is a rare, difficult-to-diagnose clinical condition that presents as acute abdomen and often leads to significant morbidity due to the delay in diagnosis and surgical treatment. Since first reported by Wendel in 1898 as a “floating gallbladder,” more than five hundred cases have been reported in the literature with an incidence of approximately 1 in 365,000 hospital admissions [1, 2]. Volvulus of the gallbladder usually presents in elderly frail women in their seventh or eighth decade of life [3]. With prompt surgical intervention, the potential for gallbladder gangrene and perforation can be averted and an excellent prognosis is achieved [4–6]. Choledochal cysts are also uncommon pathological dilations of the biliary tree, first reported in [7]. While being associated with multiple congenital anomalies of the hepatobiliary and pancreatic systems, they have not been reported to increase the likelihood of gallbladder volvulus. Herein we describe a case of successful surgical treatment of gallbladder volvulus in an elderly woman with concomitant Todani type IC choledochal cyst, shedding the light on the perioperative management of an extremely rare biliary tree disease combination [8].

## 2. Case Presentation

**Figure 1:**
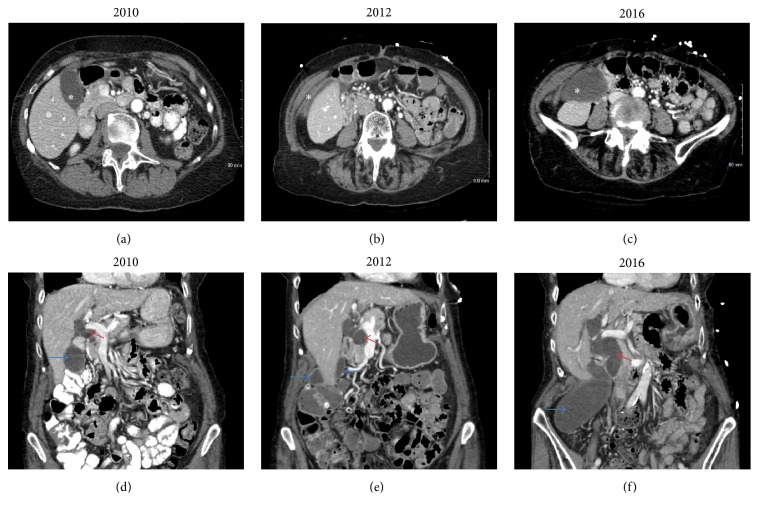
(a), (b), and (c) show computed tomography axial images of the abdomen with the gallbladder marked by (*∗*). Notice the different anatomical positions of the gallbladder over a six-year period. (d), (e), and (f) show computed tomography coronal images of the abdomen showing changes in the anatomical position of the gallbladder (blue arrow); the choledochal cyst (red arrow) is shown to be increasing in size over the same period.

**Figure 2:**
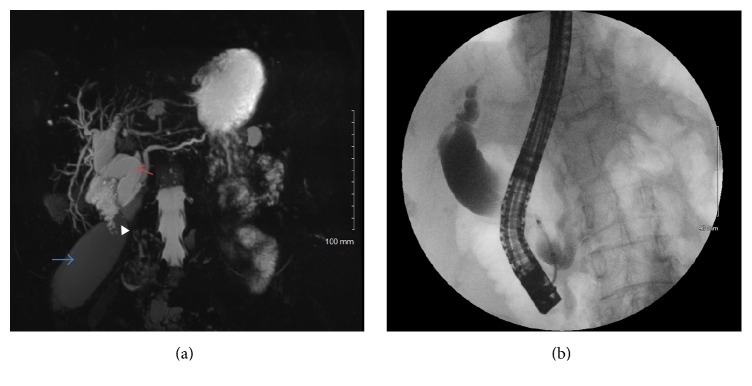
An MRCP image is shown in (a), demonstrating the type 1C choledochal cyst (red arrow), in addition to the distended, torsed gallbladder (blue arrow). Notice the severe narrowing of the gallbladder infundibulum as it joins the dilated bile duct (arrowhead). (b) is an ERCP cholangiogram demonstrating the choledochal cyst without distal common bile duct stricture or stone. The gallbladder did not opacify with contrast due to cystic duct obstruction.

A 92-year-old woman presented to the emergency room with acute onset of right upper quadrant pain, nausea, and vomiting. She was previously in her usual state of health with minimal medical or surgical comorbidities with the exception of a minor weight loss. Her past medical history is significant for gastroesophageal reflux, history of peptic ulcer disease, and osteoarthritis. She has had cataract surgery in the past in addition to colonic polypectomy for benign polyps a few years prior to her presentation. Her family history was significant for uterine cancer in her mother, without known genetic predisposition. On physical examination, she was hemodynamically normal and the only pertinent finding was tenderness to palpation in her right upper and lower abdominal quadrants. All blood tests were within normal limits including a white blood cell count and liver function tests. A right upper quadrant ultrasound demonstrated gallbladder distention with mild gallbladder wall thickening, but no evidence of gallstones, sludge, or pericholecystic fluid. A computed tomography (CT) scan demonstrated a markedly dilated extrahepatic biliary ductal system (intrapancreatic common bile duct > 2.5 cm) and a markedly distended gallbladder (Figure 1). The patient was admitted to the hospital for treatment of acute cholecystitis including bowel rest and antibiotics. Given the marked dilation of the intra- and extrahepatic bile ducts an obstructing mass at the head of the pancreas or the distal bile duct had to be ruled out. Magnetic resonance cholangiopancreatography (MRCP) demonstrated a distended gallbladder with wall thickening, edema, and pericholecystic fluid confirming acalculous cholecystitis, with a mention of focal narrowing and wall thickening of the cystic duct. The extrahepatic bile duct had a fusiform dilation measuring 27 mm in the largest dimension (Figure 2). Endoscopic retrograde cholangiopancreatography (ERCP) and an endoscopic ultrasound (EUS) failed to show a pancreatic head mass or distal common bile duct stricture; however the cystic duct did not opacify, suggesting an obstruction in the absence of gallstones. The patient's clinical status did not improve during a short period of observation; she developed increasing abdominal pain and leukocytosis to 13.5 e^3^/*μ*L. After reviewing of previous CT scans spanning several years, the gallbladder was noted to be located in several different locations adding suspicion for gallbladder torsion to the differential diagnosis (Figure 1). The patient was consented for open cholecystectomy due to the extremely large size of her gallbladder, her concomitant small body habitus, and a relative delay in diagnosis.

**Figure 3:**
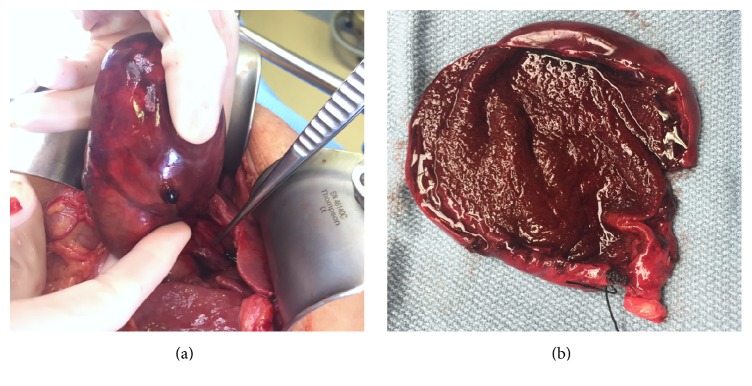
An intraoperative photograph showing the severe distention and discoloration of the gallbladder. This was observed upon entry into the abdomen, the gallbladder was found to be torsed around the cystic duct and cystic artery (shown next to the instrument tip), and there was no other attachment between the gallbladder and the liver.  Figure 2(b) shows the gallbladder after it was opened on the back table; no gallstones were found. There was a significant gallbladder wall thickening and necrosis.

At laparotomy, a gangrenous, necrotic gallbladder was identified in the right lower quadrant and was nonadherent to the liver bed. The gallbladder was completely torsed (360 degrees) around the cystic duct and cystic artery. The gallbladder was so mobile that it could be brought out onto the abdominal wall and detorsed; a blood clot was visible in the gallbladder mesentery at the point of torsion. The gallbladder was readily removed following simple ligation of the cystic artery and duct (Figure 3). The choledochal cyst was not addressed given her advanced age and lack of malignancy seen on axial imaging, ERCP, and EUS. Inspection of the specimen revealed significant gallbladder wall thickening and absence of gallstones (Figure 3). The patient recovered without major events and was discharged to a rehabilitation facility on postoperative day four. Final pathology revealed severe acute cholecystitis with transmural necrosis and acute serositis.

## 3. Discussion

Gallbladder volvulus or torsion occurs when the gallbladder rotates either clockwise or counterclockwise around its mesentery along the axis of the cystic duct and cystic artery causing complete obstruction of blood flow and biliary drainage resulting in acute gangrenous cholecystitis [3]. This condition is extremely rare; it occurs in less than 0.1% patients who undergo urgent cholecystectomies for presumed acute cholecystitis [4]. The incidence of gallbladder volvulus increases with age and peaks in subjects between sixty and eighty years of age. It is more common in women, with a female : male ratio of 3-4 : 1 [2].

The etiology of gallbladder torsion is unknown. Anatomic anomalies, such as an abnormally long mesentery or abnormal fixation of the gallbladder to the liver, can result in a suspended gallbladder allowing it to freely float from the liver bed [3]. It is thought to occur more commonly in the elderly due to the loss of visceral fat, liver atrophy, and increased elasticity that allows the gallbladder to freely hang or “float.” Significant peristaltic movements of the stomach, duodenum, and nearby colon, in addition to mechanical predisposition caused by kyphoscoliosis, have been cited in the literature as predisposing factors for gallbladder torsion [9, 10]. Gallstones are considered incidental rather than causative when found in patients with gallbladder volvulus. They are only present in 25–50% of patients with gallbladder torsion, and thus they are unlikely to be the underlying etiology [10].

Patients with gallbladder torsion present with symptoms similar to biliary colic or acute cholecystitis including acute onset of right upper quadrant pain. The acuteness of the presentation depends on whether the volvulus is partial or complete; a partial torsion is followed by spontaneous detorsion and resolution of symptoms, thus presenting like biliary colic, whereas a complete torsion (typically of more than 180°) presents with acute cholecystitis-type symptoms with worsening over the ensuing hours to days [11]. As in our patient, laboratory investigations typically show a normal to elevated white blood cell count. Liver function tests are normal as the common bile duct remains unobstructed [9]. Preoperative diagnosis of gallbladder torsion is difficult since clinical features overlap with other acute gallbladder conditions [12]. The distinction between torsion and acute calculous cholecystitis is important; while cholecystitis can initially be treated conservatively, delay in intervention could prove fatal in the setting of gallbladder torsion. Torsion of the gallbladder has a mortality rate of 6%; none of the reported deaths occurred in patients diagnosed preoperatively [13]. Thus, early diagnosis and prompt intervention can reduce the mortality associated with this condition [13–15].

In the past, gallbladder torsion was typically diagnosed intraoperatively. However, advancements in diagnostic imaging as well as the increased use of imaging in the workup of patients with acute abdomen have led to an increase in preoperative diagnosis [16]. Ultrasonography and computed tomography are the main imaging modalities used for diagnosis, although they are often nonspecific [12]. These imaging approaches may reveal a floating gallbladder without stones, nonadherent to the liver bed, and outside of the usual anatomic fossa [3]. Previous reports have identified computed tomography criteria for identifying gallbladder torsion including (1) fluid collection between the gallbladder and its fossa, (2) the presence of horizontal rather than vertical axis of the gallbladder, (3) the presence of a cystic duct located on the right side of the gallbladder, and (4) the “whirl sign” of a twisted cystic artery with medial deviation of the extrahepatic bile duct [17]. A hepatobiliary iminodiacetic acid (HIDA) scan theoretically shows a characteristic, though not sensitive, “bull's eye” appearance of the torsed gallbladder [18]. T2-weighted MRI images are useful for evaluating necrosis of the gallbladder wall as in cases of torsion [19, 20]. More recent reports have suggested the use of chronological and sequential diagnostic imaging as a means of diagnosing gallbladder torsion [21]. The present case illustrates the usefulness of comparing imaging from prior admissions in order to make the preoperative diagnosis of gallbladder torsion, which can be appreciated retrospectively (Figure 1). Computed tomography axial images from four and six years priorly illustrate the mobile and floating nature of our patient's gallbladder; these can be helpful when patients present with atypical symptoms, poor response to antibiotics, or acalculous cholecystitis.

Early diagnosis and prompt surgical intervention with detorsion and cholecystectomy are important to avoid complications of perforation and bilious peritonitis [22]. The use of transhepatic cholecystostomy tube for management of gallbladder volvulus is not recommended due to the extrahepatic location of the gallbladder and the necrotic gallbladder wall, both of which increase the likelihood of bile spillage and peritonitis. While laparoscopic detorsion and cholecystectomy is effective in the management of gallbladder torsion, we chose open cholecystectomy due to the very large size of the gallbladder reaching a pelvic location in a patient with an extremely small body habitus (no room for laparoscopic instrumentation/visualization) [23, 24]. Laparoscopic cholecystectomy and dissection of the critical view of safety in gallbladder torsion patients id usually straightforward given the lack of a cystic plate attaching the gallbladder to the liver [12, 23].

To our knowledge, this is the first report in the literature that describes the presence of gallbladder torsion in a patient with a choledochal cyst. Choledochal cysts have been associated with other anomalies within the pancreaticobiliary tree including multiseptated gallbladder, heterotopic pancreas, and pancreatic divisum, but they have not been reported to predispose to volvulus of the gallbladder [8, 25]. Choledochal cysts are classified into five types based on the Alonso-Lej modification of the initial Todani classification [26]. Our patient had a type I choledochal cyst which is the most common cyst type [7]. The presence of a choledochal cyst is considered an indication for surgical resection as many of these become symptomatic, presenting with recurrent cholangitis or pancreatitis and harbor a risk of synchronous cholangiocarcinoma [27, 28]. In a large, multi-institutional western study that included 394 patients, cyst excision to include extrahepatic bile duct resection and hepaticoenterostomy was the standard of surgical care and completed in 80.3% of patients [7]. Malignancy was found in 9.1% and at follow-up, 3.3% of patients had cancer recurrence with a median survival of 3.5 years [7]. This study reconfirmed prophylactic surgical resection of choledochal cysts.

**Figure 4:**
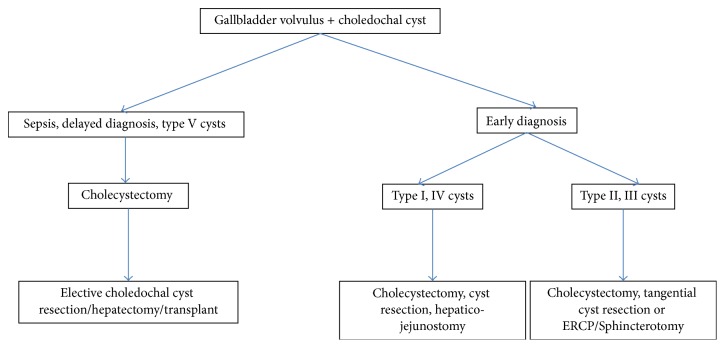
Algorithm for treatment of gallbladder volvulus in patients with concomitant choledochal cysts.

Due to the rarity of this combination of gallbladder torsion and choledochal cyst, a hepatobiliary surgeon should be involved from the outset. Prompt gallbladder detorsion and cholecystectomy is the standard of care for gallbladder volvulus regardless of the timing of diagnosis (pre- or intraoperatively). Extrapolation from common surgical practice precludes excision of the choledochal cyst and construction of a hepaticoenterostomy in the setting of an acutely inflamed and potentially infected field; thus laparoscopic cholecystectomy should be done first. The patient can then be brought back electively for choledochal cyst resection. In cases of acute cholecystitis without gallbladder torsion, conservative management of cholecystitis can be attempted with antibiotics with or without percutaneous cholecystostomy drainage which may then allow for a one-stage procedure (cholecystectomy and choledochal cyst resection). We suggest an algorithm extrapolated from literature data suggesting a plan of action when a gallbladder volvulus is encountered in a patient with choledochal cysts (Figure 4). It is based on the patient's clinical status and the chronicity of the volvulus in addition to the cyst type, as the procedures needed to treat choledochal cysts vary from a simple ERCP/sphincterotomy in choledochoceles (Todani type III cysts), reaching liver transplant in Caroli's disease (Todani type V cysts).

## 4. Conclusion

Gallbladder volvulus and choledochal cysts are two very rare entities of the biliary tree. Volvulus should be considered in the elderly female patients with symptoms suggestive of sudden onset of acute acalculous cholecystitis with imaging characteristics pointing towards a free-floating gallbladder. While gallbladder volvulus requires a high index of suspicion and early surgical intervention to avoid significant morbidity and mortality in a frail patient population, choledochal cysts can be dealt with electively taking into account their symptoms and risk for malignancy relative to age.
